# Probing the mechanism of nick searching by LIG1 at the single-molecule level

**DOI:** 10.1093/nar/gkae865

**Published:** 2024-10-15

**Authors:** Surajit Chatterjee, Loïc Chaubet, Aafke van den Berg, Ann Mukhortava, Danah Almohdar, Jacob Ratcliffe, Mitchell Gulkis, Melike Çağlayan

**Affiliations:** Department of Biochemistry and Molecular Biology, University of Florida, 1200 Newell Drive, Gainesville, FL 32610, USA; LUMICKS B.V., 1059 CH, Paalbergweg 31105 AG, Amsterdam, The Netherlands; LUMICKS B.V., 1059 CH, Paalbergweg 31105 AG, Amsterdam, The Netherlands; LUMICKS B.V., 1059 CH, Paalbergweg 31105 AG, Amsterdam, The Netherlands; Department of Biochemistry and Molecular Biology, University of Florida, 1200 Newell Drive, Gainesville, FL 32610, USA; Department of Biochemistry and Molecular Biology, University of Florida, 1200 Newell Drive, Gainesville, FL 32610, USA; Department of Biochemistry and Molecular Biology, University of Florida, 1200 Newell Drive, Gainesville, FL 32610, USA; Department of Biochemistry and Molecular Biology, University of Florida, 1200 Newell Drive, Gainesville, FL 32610, USA

## Abstract

DNA ligase 1 (LIG1) joins Okazaki fragments during the nuclear replication and completes DNA repair pathways by joining 3′-OH and 5′-PO_4_ ends of nick at the final step. Yet, the mechanism of how LIG1 searches for a nick at single-molecule level is unknown. Here, we combine single-molecule fluorescence microscopy approaches, C-Trap and total internal reflection fluorescence (TIRF), to investigate the dynamics of LIG1-nick DNA binding. Our C-Trap data reveal that DNA binding by LIG1 full-length is enriched near the nick sites and the protein exhibits diffusive behavior to form a long-lived ligase/nick complex after binding to a non-nick region. However, LIG1 C-terminal mutant, containing the catalytic core and DNA-binding domain, predominantly binds throughout DNA non-specifically to the regions lacking nick site for shorter time. These results are further supported by TIRF data for LIG1 binding to DNA with a single nick site and demonstrate that a fraction of LIG1 full-length binds significantly longer period compared to the C-terminal mutant. Overall comparison of DNA binding modes provides a mechanistic model where the N-terminal domain promotes 1D diffusion and the enrichment of LIG1 binding at nick sites with longer binding lifetime, thereby facilitating an efficient nick search process.

## Graphical Abstract

**Graphical Abstract:**
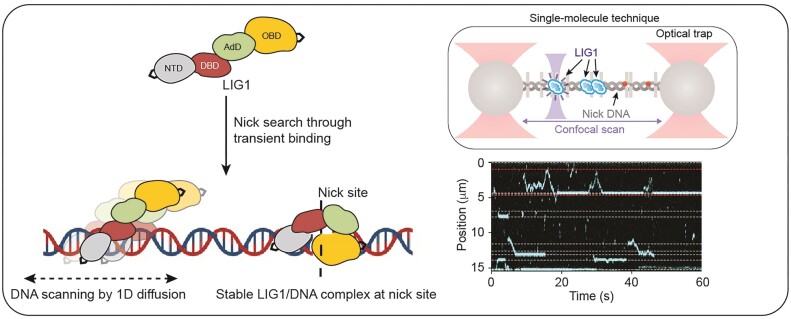

## Introduction

Genomic DNA is susceptible to damage from numerous sources, including endogenous and environmental factors (1,2). One potentially toxic form of DNA damage is the DNA strand breaks that occur naturally as intermediates during almost all cellular DNA transactions such as replication, repair and recombination (3). If left unrepaired and allowed to persist, these strand breaks could result in potentially deleterious nicks in the DNA backbone, making them vulnerable to exonuclease-mediated degradation of DNA ends, leading to an increased frequency of recombination, and the formation of lethal double-strand breaks (4). The interruptions in the phosphodiester backbone of DNA are repaired by DNA ligases that convert nicks into phosphodiester bonds during discontinuous DNA synthesis on the lagging strand of the replication fork and at the final step of most DNA repair pathways (5). Therefore, the nick sealing by DNA ligase is critical to maintain overall genome integrity (6,7).

All higher eukaryotic DNA ligases utilize ATP as a cofactor for joining two adjacent 5′-phosphate (PO_4_) and 3′-hydoxyl (OH) ends of nick in the energetically favorable and consecutive three-step of the ligation reaction (3–6). During the first two steps, an adenosine 5′-monophosphate (AMP) moiety is covalently bound to an active site lysine residue of the ligase leading to the formation of DNA ligase-AMP intermediate (step 1), and then the AMP is transferred from the ligase to the 5′-PO_4_ end of a nick (step 2) resulting in the formation of the adenylated form of DNA (8,9). This DNA-AMP intermediate further activates the 5′-PO_4_ on the downstream strand for a nucleophilic attack by the upstream 3′-OH that displaces the adenylate moiety and catalyzes a phosphodiester bond formation between DNA ends during the final step 3 of the ligation reaction (10). Several enzymes that catalyze nucleotidyl transfers such as DNA ligases (ATP- and NAD^+^-dependent), RNA ligases and RNA capping enzymes share this conserved ligation chemistry (11,12). Although the nick sealing reaction is highly conserved and ultimate step of nuclear replication and DNA repair, how a DNA ligase recognizes and binds to its target nick substrate remains a critical knowledge gap in our understanding the mechanism of DNA ligation.

The possible mechanism of nick binding has been suggested for DNA ligases from small viral DNA ligases, such as Vaccinia and Chlorella viruses (13–19). DNA ligase adenylation at the active site lysine residue during the initial step of the ligation reaction is rapid and stable in the absence of nick DNA, which suggests that most ligase molecules in the cells exist in this initial adenylated state (20). Furthermore, the requirement of a phosphate group at the 5′-end for proper nick sealing and weaker DNA nick binding by non-adenylated eukaryotic DNA ligase have been reported (15,16), suggesting that the initial formation of DNA ligase-AMP intermediate is a prerequisite for nick recognition (21). The crystal structures of both NAD^+^- and ATP-dependent DNA ligases from various sources such as T4 DNA ligase, *Escherichia coli*, *Thermus thermophilus*, *Pyrococcus furiosus*, mycobacteria and *Saccharomyces cerevisiae* have revealed a conserved mechanism that involves the distortion of the DNA helix upon nick binding which causes the region upstream of the nick to compress into an A-form helical conformation (22–29). Regarding the dynamics of nick sealing, the real-time fluorescence measurement of the ligation reaction using the TFAM reporter substrate with the T4 bacteriophage ligase demonstrated the first evidence of a multi-step DNA binding and catalysis, suggesting a transition between two different conformational states of DNA ligase/nick substrate complex (23).

Human DNA ligases, DNA ligase I, IIIα and IV, share a common catalytic core consisting of the oligonucleotide binding-fold (OB-fold) domain (OBD) and adenylation domain (AdD) that are conserved in other DNA ligases and nucleotidyl transferases including RNA ligases and mRNA capping enzymes (4–6). The catalytic activity of the enzyme, both for ATP- and NAD^+^-dependent DNA ligases, is largely governed by the active site residues residing in the AdD and the OBD domains (20). Next to this catalytic core, the DNA-binding domain (DBD) has been identified in the DNA ligases of higher eukaryotes (20).

Human DNA ligase 1 (LIG1), the main replicative ligase, carries out the nick sealing for Okazaki fragment maturation that occurs thousands to millions of times during each DNA replication cycle in eukaryotic cells (30). In addition, LIG1 joins the broken phosphodiester bonds to create an uninterrupted DNA strand by sealing a final nick product at the ultimate step after the excision of a damaged base and subsequent re-synthesis of DNA during DNA repair pathways (31–33). Notably, the non-synonymous mutations (P529L, E566K, R641L and R771W) in the *LIG1* gene have been described in the patients with LIG1 deficiency syndrome exhibiting immunodeficiency and cancer predisposition as well as aberrant DNA repair and replication (34,35).

The first structure of LIG1 determined by X-ray crystallography revealed a model of nick binding mechanism mediated through three domains of the ligase (36). According to this model, the catalytic region adopts extended and asymmetric conformation in the absence of DNA and a large conformational change occurs between AdD and OBD domains that interact with the DBD to form a ligase protein clamp-like architecture that encircles a nick (36). Furthermore, the most extensive protein–DNA contacts occur mainly in the minor groove and are made by the flat surface of the DBD through 12 α-helical extensions, which provides a platform for nick binding and stimulates enzymatic activity of the catalytic core for catalysis (36). Further structural studies of LIG1-nick DNA complexes revealed the mechanism by which the ligase active site maintains faithful nick sealing by discriminating unusual DNA ends (37–40). For example, it has been shown that glutamic acids residues, Glu346 and Glu592, reinforce high fidelity that is mediated by a catalytic Mg^2+^-dependent DNA binding during the adenyl transfer and nick sealing steps of the ligation reaction (37). In our studies, we reported how LIG1 engages with the nick repair intermediate containing canonical, mismatched or damaged ends after an incorporation of non-canonical mismatch or oxidatively damaged (8oxodGTP) nucleotide by DNA polymerases during the downstream steps of the repair pathway (41–47). In our LIG1 structures, we also demonstrated that LIG1 active site engages with nick containing non-canonical ends distinctly and deters (A:C) or favors (G:T) mismatches depending on the architecture of 3′-primer/template base pair (38). Furthermore, our recent LIG1 structures revealed that the processing of nicks with oxidatively DNA damaged ends relies on the interaction network of the critical side chains around nick site depending on dual coding potential of 8oxodGTP(*anti*):C(*anti*) and 8oxodGTP(*syn*):A(*anti*) that forms non-mutagenic Watson-Crick and mutagenic Hoogsteen base pairing, respectively (39). We also uncovered that LIG1 can efficiently seal almost all non-canonical nick DNA substrates containing a single ribonucleotide at the 3′-end that mimic the products of ribonucleotide incorporation by repair or replication DNA polymerases (45). Our recent LIG1/DNA-RNA heteroduplex structures demonstrated that LIG1 lacks a discrimination against ‘wrong’ sugar at the 3′-end of nick and the ligase active site can accommodate ribonucleotide-containing ends during third step of the ligation reaction where a final phosphodiester bond is formed (40). Although these structure/function studies of LIG1 provided insights into the modular domain architecture of the ligase and how the ligase surveils unusual DNA ends at nick through interactions with Mg^2+^ ion and active site residues during three-step of ligation reaction, the dynamics of ligation reaction and the mechanism of the nick searching by LIG1 have not been elucidated at the single-molecule level.

In addition to the conserved catalytic core, LIG1 contains a distinct N-terminal domain including a proliferating cell nuclear antigen (PCNA) interacting peptide (PIP) box and a nuclear localization signal (NLS) that direct the ligase to participate in nuclear replication (48,49). This unstructured and non-catalytic region also mediates LIG1 interactions with other repair and replication proteins such as PCNA, clamp loader replication factor A (RFA) and C (RFC), Rad9-Rad1-Hus1 (9, 1, 1) complex, a heterotrimeric clamp involved in cell cycle checkpoints, as well as DNA polymerase β, the main base excision repair polymerase (50–54). The studies reported conflicting results regarding the contribution of this unstructured region to catalytic activity of LIG1 depending on reaction conditions (48–51). Yet, the role of N-terminal domain of LIG1 that is not visible in X-ray and cryo-EM structures for nick sealing mechanism is a significant gap in our understanding of the ligation reaction.

In the present study, we investigated how LIG1 searches for a nick at the single-molecule level. Comparing DNA-binding modes of the full-length and C-terminal mutant of LIG1, we also questioned the role of the N-terminal domain for an efficient nick searching mechanism. For this purpose, we used a combined approach of the C-Trap (LUMICKS) and total internal reflection fluorescence (TIRF) imaging to precisely visualize the positions of fluorescently labeled LIG1 on a biotinylated DNA substrate containing defined nick sites. Our findings revealed 1D diffusion mode of DNA binding and formation of long-lived LIG1-DNA complex at a nick site. We showed a stable nick binding by LIG1 full-length compared to a significantly shorter binding by LIG1 C-terminal mutant on the DNA containing a single nick site. Furthermore, using DNA without a nick, our results demonstrated very fast binding events by LIG1 full-length. Overall, our single-molecule characterization of LIG1-nick DNA binding dynamics provides a novel insight into the mechanism driving nick search by LIG1 and the role of non-catalytic N-terminal region in reducing non-nick site binding on the DNA and facilitating 1D diffusion mode that leads to effective and more specific nick search.

## Materials and methods

### Purification of DNA ligase 1

For single-molecule characterization of the ligase-nick DNA binding, we used human DNA ligase 1 (LIG1) proteins coding full-length (1–919 amino acids) and C-terminal (261–919 amino acids) region harboring the AdD, OBD and DBD domains (Supplementary Figure S1A). The recombinant LIG1 proteins with 6x his-tag (pET-24b) were overexpressed in *E. coli* (DE3) cells and grown in Terrific Broth (TB) media with kanamycin (50 μgml^−1^) and chloramphenicol (34 μgml^−1^) at 37°C as described (38–47). Once the OD_600_ reached 1.0, the cells were induced with 0.5 mM isopropyl β-D-thiogalactoside (IPTG) and overexpression was continued overnight at 20°C. After centrifugation, the cells were lysed in the lysis buffer containing 50 mM Tris-HCl (pH 7.0), 500 mM NaCl, 20 mM imidazole, 2 mM β-mercaptoethanol, 5% glycerol and 1 mM phenylmethylsulfonyl fluoride (PMSF) by sonication at 4°C. The lysate was pelleted at 31000 × *g* for 90 min at 4°C. The cell lysis solution was clarified and then loaded onto a HisTrap HP column in the binding buffer containing 50 mM Tris-HCl (pH 7.0), 500 mM NaCl, 20 mM imidazole, 2 mM β-mercaptoethanol and 5% glycerol. The protein was then eluted with an increasing imidazole gradient (0–500 mM) at 4°C. The collected fractions were subsequently loaded onto a HiTrap Heparin in the binding buffer containing 20 mM Tris-HCl (pH 7.0), 50 mM NaCl, 2 mM β-mercaptoethanol and 5% glycerol, and then eluted with a linear gradient of NaCl up to 1 M. LIG1 proteins were further purified by Superdex 200 10/300 column in the buffer containing 50 mM Tris-HCl (pH 7.0), 200 mM NaCl, 1 mM dithiothreitol (DTT) and 5% glycerol. All proteins concentrated, frozen in liquid nitrogen and stored at −80°C. Purity and concentrations of the resulting protein solutions were confirmed by sodium dodecyl sulfate-polyacrylamide gel electrophoresis gel analysis and absorbance at 280 nm.

### Fluorescent labeling of LIG1

For single-molecule experiments in the C-Trap, we fluorescently labeled LIG1 full-length and C-terminal proteins by amine reactive NHS ester-conjugated Alexa Fluor (AF)488 (LIG1^AF488^) according to the manufacturer’s instructions (Thermo Fisher). For single-molecule TIRF imaging, we fluorescently labeled LIG1 full-length and C-terminal proteins by Amersham Cy5 monoreactive NHS ester dye (LIG1^Cy5^) pack (Cytiva) as previously reported (55). Labeling efficiencies were finally determined by calculating the molar ratio of dye to protein by measuring absorbance at 280 and 488 nm (LIG1^AF488^), and 649 nm (LIG1^Cy5^) and observed between approximately 1.0–2.0 labels per protein (Supplementary Table S1). We then pre-adenylated LIG1^AF488^ and LIG1^Cy5^ by incubating the proteins in the buffer containing a final concentration of 10 mM ATP and then dialyzed against the protein storage buffer. Single-use aliquots of fluorescently labeled and pre-adenylated LIG1 proteins were flash-frozen with liquid nitrogen and stored until use at −80°C.

### DNA ligation assays

Nick DNA substrate including preinserted 3′-dG:C and a 6-carboxyfluorescein (6-FAM) label (Supplementary Table S2) was used to evaluate the ligation efficiency of LIG1 proteins (full-length and C-terminal) after labeling (Supplementary Figure S1B). The reaction containing 50 mM Tris-HCl (pH: 7.5), 100 mM KCl, 10 mM MgCl_2_, 1 mM ATP, 1 mM DTT, 100 μgml^−1^ BSA, 1% glycerol and nick DNA substrate (500 nM) was initiated by the addition of LIG1 (100 nM). The reaction samples were incubated at 37°C, stopped by quenching with an equal amount of the buffer containing 95% formamide, 20 mM ethylenediaminetetraacetic acid, 0.02% bromophenol blue and 0.02% xylene cyanol, and collected at the time points indicated in the figure legends. The reaction products were then separated by electrophoresis on an 18% denaturing polyacrylamide gel. The gels were scanned with a Typhoon Phosphor Imager (Amersham Typhoon RGB), and the data were analyzed using ImageQuant software as reported before (38–47). Using this ligation assay, we confirmed the nick sealing efficiencies of LIG1 full-length and C-terminal proteins and observed fully active enzymes after fluorescently labeling (Supplementary Figure S1C,D).

### Nick DNA substrates used in the single molecule assays

For single molecule characterization of LIG1-DNA binding in the C-Trap, we used a custom-generated biotinylated double-stranded (ds) lambda DNA (48.5 kbp) with ten methylguanine lesions (nicks) at the defined locations as well as two fluorophores (ATTO647N) at positions 33 786 and 44 826 bp (Supplementary Figure S2A). DNA substrate was held between two 4.89 μm streptavidin-coated polystyrene bead (0.1% w/v) handles (Supplementary Figure S2B,C) for the C-Trap experiments. For single-molecule characterization of LIG1-DNA binding in the TIRF, we used 5′-biotinylated dsDNA substrate with a AF488 label at the 3′-end (Figure 4A). The sequence information for the DNA substrates containing either a single nick at the defined position with and without 3′-ddC modification or no nick site are presented in Supplementary Table S3.

### Single-molecule analyses of LIG1 binding to nick DNA in the C-trap

We performed single-molecule experiments in the C-Trap which combines a confocal fluorescence microscopy with dual-trap optical tweezers (LUMICKS) as described (56). The microfluidic flow-cell containing four distinct flow channels separated by laminar flow was moved by a computer-controlled stage to allow two optical traps to traverse the different laminar layers (Supplementary Figure S2B). The single-molecule ligase-nick DNA binding experiments were performed in the buffer containing 1 mM HEPES (pH 7.4), 20 mM NaCl, 0.02% bovine serum albumin (BSA) (w/v) and 0.002% Tween 20 (v/v). DNA substrate was diluted to a final concentration of 1.16 pM, and LIG1^AF488^ protein was added to the mixture to a final concentration of 0.1–0.2 nM. During experiments, a single DNA tether is suspended between two polystyrene beads trapped by the optical tweezers to directly visualize the dynamics of nick DNA binding by AF^488−^labeled LIG1. When a single DNA tether captured between two beads was confirmed, we moved the traps into the protein channel and began confocal scanning to observe real-time LIG1 binding to the DNA.

The flow cell was first passivated with 0.05% (w/v) casein in PBS by flushing for 20 min and then incubating for an additional 10–20 min. The flow cell was then rinsed with the buffer for 20 min. For data collection, channels 1 and 2 were filled with 4.89 μm streptavidin-coated polystyrene beads and biotinylated lambda dsDNA that were diluted to 0.005% w/v and 0.05 ng/μl in the buffer, respectively. Channel 3 was used for trap calibration and channel 4 contained LIG1^AF488^ protein in the same buffer. Before starting the experiment, the protein channel was flushed for 10 min, incubated with LIG1 for another 30 min, and then rinsed with the buffer. While maintaining flow at a constant pressure of 0.3 bar, single beads were caught in both optical traps in channel 1 using a trap stiffness of 0.25 pN/nm. Then, the beads were moved to channel 2 for DNA capture. To tether DNA between the two traps, the bead in the left trap (upstream) was held in a constant position while the bead in the right trap (downstream) was moved closer and further away from the left bead. A characteristic increase in the force with increasing distance between the beads indicated the attachment of a DNA tether. A force-distance curve was measured for every tether and compared to the extensible wormlike chain model for dsDNA to verify that a single tether of dsDNA was caught. Once confirmed, the traps were moved to channel 4, and confocal line scanning of the nick DNA began. The Atto647N fluorophores on the DNA were excited intermittently to extend the lifetime of the fluorophores while AF^488^ was continuously excited to monitor the ligase-nick DNA binding events.

Plotting the 1D confocal line scans as a function of time produces kymographs which show the position of AF^488−^labeled LIG1 molecules as a function of time along the nick DNA. We located the markers using a peak detection algorithm on the red channel and then used the known coordinates of the markers to identify the locations of the nicks. The construct was imaged using the integrated confocal system at 40 ms time resolution. During experiments in the C-Trap, the markers are visible as fluorescent, red spots on the confocal images. During analysis, we locate the positions of the red spots using a Gaussian fit and note down the coordinates of markers (in microns). With that we learn the relation between bp position of markers and their positions in um along the stretched DNA on the fluorescent image. Furthermore, during the analysis, we can locate the binding events along DNA in μm and determine the bp position of the binding events with the known conversion μm to bp for markers.

#### Data analysis and calculation of diffusion coefficients and unbinding times

Data were analyzed with customer software from the LUMICKS. The tracking of LIG1-nick DNA binding events on kymographs was performed using the Lakeview, and analysis was performed using Pylake. Confocal scans and force spectroscopy data were exported from the LUMICKS Bluelake acquisition software and processed using custom-written python scripts utilizing the Pylake package (v1.3.0 https://zenodo.org/records/10283787). Since the DNA tether is narrower than the pixel size of the confocal microscope, we employed a 1D line scan which allowed faster frame rates than 2D scanning. Binding density profiles were calculated by averaging over time the intensity of binding events of a kymograph. The coordinates of the nicks were computed by utilizing the known coordinates of the ATTO647N dyes. Profiles of multiple kymographs were combined by interpolating individual profiles and then adding the interpolated profiles together. For binding profiles of LIG1 full-length protein, 3 kymographs were used, while we used 5 kymographs for binding profiles of LIG1 C-terminal protein. We used the typical kymographs that were obtained during the measurements for each protein.

The first step for determining the diffusion coefficient was to track binding events. If a track displayed both diffusive and static behavior, it was split into two parts and the diffusion coefficient was determined for each part separately. Furthermore, we excluded events that were diffusing into the beads, or were hindered by other proteins, to ensure that only tracks from proteins diffusing freely are analyzed. The diffusion coefficient was computed for each tracked event using the Covariance Based Estimator, a fast and unbiased approach for determining diffusion coefficients (57).

Unbinding times were calculated by tracking LIG1-nick DNA-binding events from start to end and extracting the duration of each tracked event. Binding durations from multiple kymographs were combined into one histogram. To determine the optimal model for the distribution of unbinding times, we fitted 1, 2, 3 and 4 exponentials using maximum likelihood fitting and computed the uncertainty of the fitted parameters using bootstrapping. We used the Bayesian information criterion (BIC) in combination with bootstrapping to determine which model was optimal (1, 2, 3 or 4 exponentials). Often, the model with the lowest BIC value is chosen as the optimal model. We noticed, however, that the bootstrapping distribution was not always unimodal for the model with the lowest BIC model, and that one of the components would be close to zero for some of the bootstrap distributions. Therefore, we selected the model that gives the lowest BIC value for which the bootstrapping distributions are still unimodal.

### Single-molecule TIRF imaging

We performed single-molecule experiments to visualize LIG1-nick DNA binding using a TIRF microscope (Nikon Eclipse Ti2-E). For this purpose, we used Cy5-labeled LIG1 (full-length and C-terminal) proteins and 3′-AF488-labeled dsDNA substrate (34-mer) including 5′-Biotin and a single or no nick site (Figure 4A). Glass coverslips (Corning) were functionalized with a mixture of biotin-PEG-SVA and mPEG-SVA (Laysan Bio, Inc.) as described previously (58). Microfluidic channels (20 μl capacity) were assembled using the passivated coverslips and Grace Bio-Labs HybriWell™ sealing system (GBL611101) and rinsed three times with the T50 buffer containing 10 mM Tris-HCl (pH 8.0) and 50 mM NaCl. Streptavidin (SA) (0.2 mg/ml) in the T50 buffer was then flowed onto the slide, reacted with the biotin-PEG for 10 min, and then washed with the T50 buffer. DNA substrate was diluted to a final concentration of 10 pM in the imaging buffer containing 1 mM HEPES (pH 7.4), 20 mM NaCl, 0.02% BSA (w/v) and 0.002% Tween 20 (v/v), flowed onto the slide, and allowed to incubate for 5 min for immobilization. Excess unbound DNA substrate was then washed by flowing 100 μl of the imaging buffer. To reduce non-specific surface binding of the labeled proteins, we further passivated the slide surface by incubating with 10 mg/ml BSA for 10 min. Oxygen-scavenging system (OSS) consisting of 44 mM glucose, 165 U/ml glucose oxidase from Aspergillus niger, catalase (2170 U/ml) as well as 10 mM Trolox were added to slow photobleaching and to reduce photo blinking, respectively. Finally, LIG1^Cy5^ proteins (1 nM) in the imaging buffer containing the OSS mixture was flowed onto the slide and allowed to equilibrate for 5 min before imaging with an objective-based TIRF microscope. We repeated the same DNA binding experiments using DNA substrate containing a single nick site in the presence of Mg^2+^ with a final concentration of 10 mM and using nick DNA substrate containing non-ligatable ends due to 3′-ddC modification. Both AF488 and Cy5 dyes were simultaneously excited using 488 and 640 nm lasers, respectively. Emissions from two fluorophores were separated into two channels using a Cairn Optosplit II image splitter and simultaneously recorded at 100 ms time resolution using a Hamamatsu SCMOS camera (77054115) using NIS-Elements acquisition software (Nikon, version: AR 6.02.01).

#### TIRF data analysis

Locations of molecules and fluorophore intensity over time traces were extracted from the raw movie files using Nikon NIS-Elements analysis software (Nikon, version: AR 6.02.01). Genuine fluorescence time traces for individual molecules were selected using the following criteria: single step photobleaching of the AF488 and at least two Cy5 intensity spikes of more than two times of the background intensity. Selected time traces were idealized using a two-state hidden Markov model (HMM) for the unbound and bound states in QuB (59). Rastergrams summarizing several individual traces were generated from the individual trace HMMs using custom written MATLAB script. From the idealized traces, dwell times of the bound and unbound states were calculated using MATLAB. Cumulative frequency of the bound and unbound dwell-time distributions was plotted and fitted in Origin Lab (version 2024b) with single or double exponential functions to obtain the bound (*t*_bound_) and unbound (*t*_unbound_) states lifetimes.

### Nick DNA-binding measurements by BioLayer interferometry assay

We measured DNA-binding kinetics of LIG1 full-length and C-terminal proteins by BioLayer interferometry (BLI) assays in real time using the Octet QKe (Fortebio). DNA-binding kinetics were performed using DNA substrate containing a single nick site and 3′-biotin label (Supplementary Table S2). SA biosensors used to attach the biotin labeled DNA were hydrated at 20°C for 20 min in the buffer containing 1 mM HEPES (pH 7.4), 20 mM NaCl, 0.02% BSA (w/v) and 0.002% Tween 20 (v/v). The sensors were then immersed in DNA (40 nM) in the kinetics buffer for 300 s. After recording an initial baseline in the kinetics buffer for 60 s, the sensors with DNA were exposed to the concentration range of LIG1 as indicated in the figure legends. DNA binding was monitored for 240 s for association step, and then in the buffer for 240 s for dissociation step. In all measurements, the affinity constants (*K*_D_), the association (*k*_on_) and dissociation (*k*_off_) rates were calculated using the ForteBio Data Analysis software with 1:1 binding model. The association rate = *k*_on_ [ligand][analyte] and the dissociation rate = *k*_off_ [ligand–analyte]. At equilibrium, forward and reverse rates are equal. All images were drawn using Origin Lab (version 2024b).

## Results

### LIG1 full-length exhibits multiple DNA binding behaviors that are enriched at nick sites

**Figure 1.**
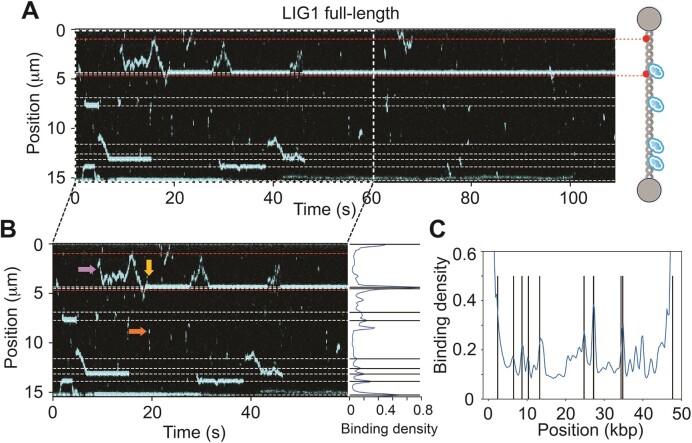
Single-molecule analysis of LIG1-nick DNA binding reveals transient non-nick binding, 1D diffusion and long-lived nick binding characteristics. (**A**) A representative kymograph shows the binding positions of AF^488^-labeled LIG1 full-length on nick DNA as a function of time. Right panel: Schematic representation of the biotinylated lambda dsDNA with two marker fluorophores (ATTO647N) and bound proteins. The DNA containing ten predicted nick sites (dashed lines) is tethered between optically trapped beads. (**B**) Selected region from the kymograph in (A) indicates different binding modes with colored arrows. Non-specific DNA binding of LIG1 is shown as an orange arrow, while 1D diffusive binding on the DNA is depicted as a pink arrow, which is followed by the formation of a static DNA-bound complex at a predicted nick site shown as a yellow arrow. Right panel: Cumulative fluorescence binding intensity profile of the full kymograph (0–555 s), where the peak intensities corresponding to bound LIG1 overlap well with the position of the predicted nick sites on the DNA (in kbp). (**C**) From the kymographs, the sum-over-time binding profile was computed by integrating all the signal over the entire length of the kymograph, then normalizing by the highest peak value. Profiles from three kymographs were combined for LIG1 full-length protein. Peaks correspond to the ligase-bound DNA and vertical lines represent the nick positions on dsDNA substrate (kbp).

We first measured LIG1-nick DNA binding at single-molecule level in the C-Trap using LIG1^AF488^ full-length and C-terminal proteins and dsDNA containing ten predicted nick sites and Atto647N fluorophores (Supplementary Figure S2). Individual kymographs demonstrated that LIG1 full-length protein can transiently bind directly to the nick site (specific binding) and to non-nick regions (non-specific binding) on the dsDNA with a mix of both static and diffusive binding behaviors (Figure 1A). Interestingly, for several binding events, a non-specifically bound LIG1 (Figure 1B, shown as orange arrow) either dissociates fast or exhibits 1D diffusive search on the DNA (Figure 1B, shown as pink arrow) followed by the formation of a static DNA-bound complex at a predicted nick site (Figure 1B, shown as yellow arrow). LIG1 can switch between 1D diffusive mode to a stable binding mode several times in the same trace, which demonstrates free interconversion between these binding modes that LIG1 exhibits during nick searching (Figure 1B, shown as pink arrow). Analysis of the overall binding density along the DNA of individual kymographs further demonstrates that LIG1 is preferentially enriched at nick locations (Figure 1C). Our results also demonstrated that LIG1 occasionally shows stable binding on a location that is not predicted nicks (Supplementary Figure S3, shown as white arrow). We also observed that diffusive ligase cannot pass static ligase (Supplementary Figure S3, shown as blue arrow) and that the LIG1 was also capable of 1D diffusion past several predicted nick sites (Supplementary Figure S3, shown as green arrow).

### Role of non-catalytic N-terminal region for nick binding by LIG1

To elucidate the role of non-catalytic N-terminal domain for LIG1 binding to nick DNA, we next used the LIG1 C-terminal protein harboring the catalytic core and DBD (Supplementary Figure S1A). We observed ∼2-fold difference in nick DNA-binding affinities of LIG1 full-length (*K*_D_: 95 nM) and C-terminal (*K*_D_: 196 nM) proteins by BLI assay (Supplementary Figure S6). To better characterize the nick DNA binding mode of both LIG1 proteins, we employed single-molecule fluorescence microscope approaches the C-Trap and TIRF.

**Figure 2.**
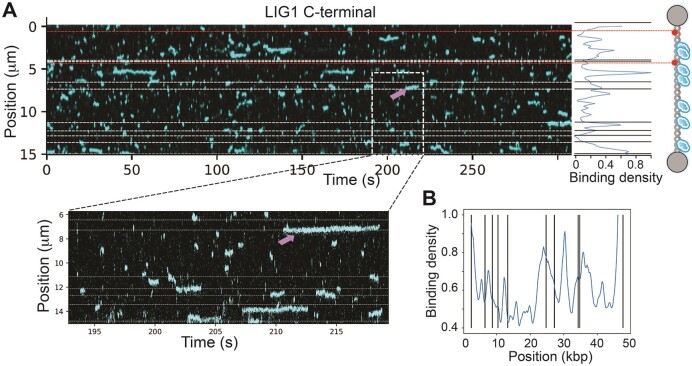
LIG1 C-terminal mutant predominantly binds to non-nick regions of the DNA for shorter periods. (**A**) The kymograph (top panel) displays single molecule dynamics AF^488^-labeled LIG1 C-terminal lacking N-terminal region of the protein binding to nick DNA. Bottom panel shows selected region of the kymograph displaying multiple non-specific off-target binding and few binding events at predicted nick sites (dashed lines). (**B**) Combined binding profiles from five kymographs for the LIG1 C-terminal protein binding exhibits no binding enrichment near the nick sites.

In the C-Trap, we observed robust binding activity throughout the DNA devoid of any specificity for the nick locations (Figure 2). LIG1 C-terminal mutant displays a single binding mode throughout the DNA, regardless of predicted nick sites. The individual kymographs showed non-preferential binding throughout the DNA and when bound at or near the nicks. In contrast to the LIG1 full-length (Figure 1), C-terminal mutant remains static. Occasionally, the LIG1 C-terminal protein seems to diffuse over a nick without switching to a static binding mode at the nick (Figure 2A, lower panel, shown as pink arrow). Overall binding density along the DNA showed little to no preferential enrichment of LIG1 C-terminal protein at nick sites (Figure 2B), suggesting that the N-terminal region plays a significant role for promoting specific binding to target nick sites by LIG1.

**Figure 3.**
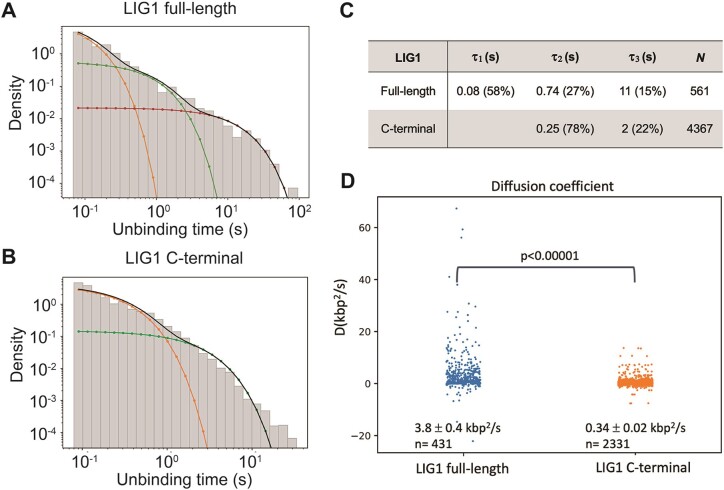
Comparisons for the unbinding times of LIG1 full-length and C-terminal proteins. (**A**and **B**) The unbinding times were fitted with three exponentials for LIG1 full-length (A) and two exponentials for C-terminal (B) time scales. (**C**) Binding lifetimes extracted by fitting 2 or 3 exponential functions to the distribution of binding lifetimes for LIG1 full-length and C-terminal proteins. *N* refers to a number of total binding events. (**D**) Distribution of the diffusion coefficients of LIG1 full-length and C-terminal proteins, estimated diffusion coefficients are determined as mean ± 1 SEM. The distributions were compared using a Welch’s T test.

To further characterize the role of the N-terminal domain, we estimated the binding lifetimes of DNA-bound LIG1 full-length and C-terminal proteins (Figure 3). The binding lifetime was defined as the lifetime of the full event, with diffusive and static binding modes combined. The binding kinetics were calculated combining the binding lifetimes of the full-length protein from six kymographs. The data can best be fit with three exponential time scales (Figure 3A and Supplementary Figure S4). For the LIG1 C-terminal protein, the binding kinetics were calculated combining binding lifetimes from three kymographs. We predominantly observed fast transient binding and the lifetimes can best be fit with two exponential time scales (Figure 3B and Supplementary Figure S5). For the LIG1 full-length (Figure 3C), we observed a frequency of 220 binding events in 10 min time scale with binding lifetimes of 0.08 s (58%), 0.74 s (27%) and 11 s (15%). This suggests that the majority of LIG1 dissociates fast, while 15% of the ligase stays bound for very long ∼11 s. For the LIG1 C-terminal (Figure 3C), we found an average frequency of 122 binding events per min with binding lifetimes of 0.25 s (78%) and 2 s (22%).

Furthermore, we calculated the diffusion coefficient for the 431 diffusive events of LIG1 full-length and the 2331 diffusive events of LIG1 C-terminal observed during our experiment (Figure 3D). We found that the rate of diffusion for LIG1 full-length (3.8 ± 0.4 kbp^2^/s) was ∼10-fold higher than that of LIG1 C-terminal (0.34 ± 0.02 kbp^2^/s), suggesting that the N-terminal domain has an important role in mediating fast and efficient diffusion of LIG1 on nick DNA. Together, overall results suggest that, without the N-terminal domain, the LIG1 C-terminal could bind non-specifically and remains bound to the DNA for significantly shorter periods compared to the full-length protein.

### Single-molecule fluorescence co-localization analyses further reveal importance of N-terminal domain of LIG1 for efficient nick searching

**Figure 4.**
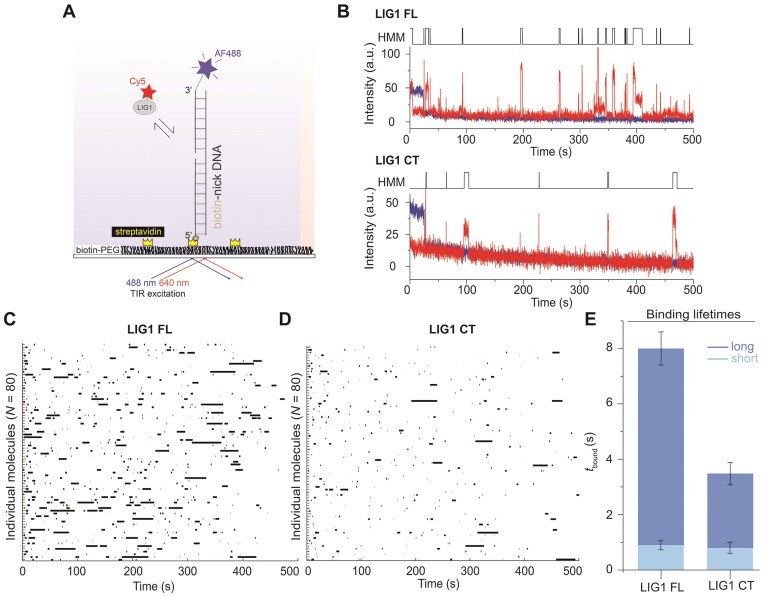
Single-molecule analyses of LIG1-nick DNA binding in the TIRF reveal longer binding for LIG1 full-length compared to the C-terminal mutant. (**A**) AF488-labeled dsDNA with a single nick were immobilized on the PEG-coated, biotinylated slide surface via biotin-SA interaction and imaged with a TIRF microscope to monitor Cy5-labeled LIG1 binding. (**B**) Fluorescence intensity versus time traces show repeated protein binding events for LIG1 full-length (FL, top panel) and the C-terminal mutant (CT, bottom panel). (**C**and **D**) Rastergrams for 80 randomly selected traces are shown for LIG1 full-length (C) and C-terminal (D) proteins displaying the distinct nick DNA binding behavior. (**E**) Difference in lifetimes of protein-bound states (*t*_bound_) are calculated from HMMs, shown on top of each trace in panel B, for LIG1 full-length and C-terminal proteins binding to the nick DNA. All values of *t*_bound_ and *t_un_*_bound_ times are presented in Supplementary Table S4.

To further characterize the LIG1-nick binding, we employed single-molecule fluorescence co-localization approach using TIRF microscopy with a AF488-labeled dsDNA containing only a single or no nick site and Cy5-labeled LIG1 full-length and C-terminal proteins. The 5′-biotinylated dsDNA was immobilized on a biotinylated glass slide utilizing the multivalency of streptavidin. Real-time LIG1^Cy5^ binding to the DNA was identified by the co-localization of AF488 and Cy5 signals within a diffraction-limited spot (Figure 4A). Analyses of individual fluorescence time trajectories show repeated transient Cy5 co-localizations with AF488, indicating dynamic binding of LIG1 on the DNA (Figure 4B). Rastergrams generated from several individual traces idealized by HMM clearly demonstrated distinct binding behaviors for the LIG1 full-length and C-terminal mutant, as indicated by the relatively fast and more frequent long-lived binding events for the full-length protein (Figure 4C,D). From the distributions of dwell times of LIG1 in the bound states from few hundred traces, we next estimated the average binding lifetimes. For the LIG1 full-length, we observed two populations with average binding lifetimes of 0.9 ± 0.17 s (70%) and 8 ± 0.6 s (30%) (Supplementary Table S4). In a control experiment, we monitored binding of LIG1^Cy5^ full-length to a DNA substrate without a nick site and observed very fast binding events with average lifetimes of 0.26 ± 0.04 s (80%) and 2.8 ± 0.5 s (20%), suggesting that LIG1 binding events occur non-specifically throughout the DNA without nick site (Supplementary Table S4). We also repeated the same DNA binding experiment using nick substrate containing non-ligatable ends due to 3′-ddC modification. Our results showed similar nick binding behavior as the unmodified and ligatable nick DNA, suggesting no nick sealing during our single-molecule experiment (Supplementary Table S5). In another control experiment, we studied the full-length LIG1 binding to the unmodified single-nick DNA in the presence of Mg^2+^ to induce nick sealing and observed binding events similar to the no-nick substrate with binding lifetimes of 0.3 ± 0.01 s (80%) and 2.9 ± 0.14 s (20%) (Supplementary Table S5). This further confirms that the long binding events observed in the absence of Mg^2+^ were for specific binding at the nick, allowing only observation of the non-specific LIG1 binding throughout the DNA. Although these TIRF measurements cannot provide diffusion information during protein binding on the DNA, together, these results clearly suggest that the long-binding events for LIG1 full-length-nick DNA interaction are due to specific binding at the nick, consistent with the C-trap data. For the LIG1 C-terminal mutant (Figure 4B,E), we observed significantly shorter binding events to DNA containing a single nick site, compared to the full-length protein, with average binding lifetimes of 0.8 ± 0.2 s (80%) and 3.5 ± 0.4 s (20%) (Supplementary Table S4). The observed unbound time was relatively higher for the C-terminal mutant (98 ± 6 s) compared to the full-length protein (42 ± 4 s). This observation is different from the C-Trap data and can be explained due to the possibility of multiple protein binding on the DNA used in the C-Trap experiments owing to its long length (48.5 kbp) compared to the short DNA (34-mer) used in the TIRF experiments. Overall, the observed binding lifetimes from single-molecule TIRF data are consistent with our C-Trap data showing that the LIG1 full-length binding events have longer lifetimes at the nick sites, whereas the C-terminal mutant binding is significantly shorter without any nick-site enrichment (Figure 4E).

**Figure 5.**
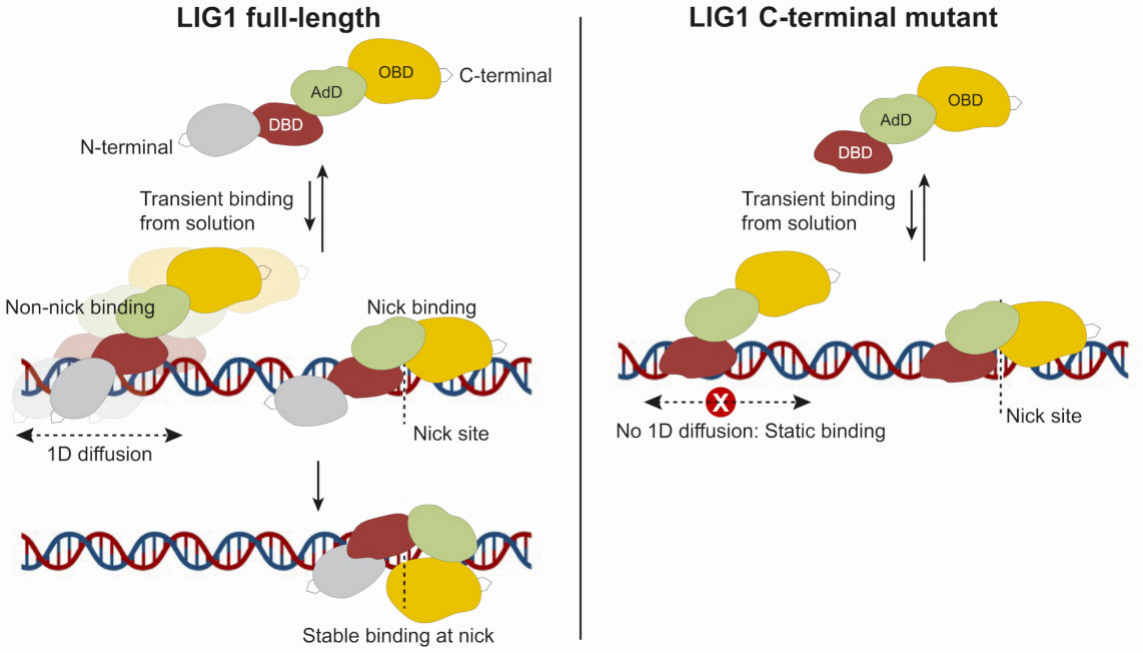
Proposed model of LIG1 nick DNA binding. Initial transient binding of LIG1 full-length protein occurs from solution. After binding, we see various types of behavior: LIG1 full-length diffuses and pauses at nick sites. Overall, the binding of LIG1 full-length is enriched at nick sites. Without the N-terminal region, the LIG1 C-terminal mutant binds non-specifically throughout the DNA only transiently without the ability to diffuse and form a stable complex at nick site.

Based on our overall results from C-Trap and TIRF, we propose a working model for nick DNA binding mode of LIG1 (Figure 5). LIG1 can initially bind to the dsDNA directly at the nick site (specific binding) as well as at non-nick regions (non-specific binding) on the DNA and the protein can diffuse to the nick site to form a stable complex after binding. Majority of the initial LIG1 full-length binding occurs through fast transient interaction, facilitating efficient nick search process. Yet only a fraction of DNA-bound LIG1 forms stably bound complex on the DNA. LIG1 without N-terminal region, however, upon initial binding non-specifically throughout the DNA, cannot diffuse along the DNA and remains bound for significantly shorter time before eventually dissociating from the DNA. Altogether, our data demonstrate the efficient mechanism of LIG1 binding to its nick site and how the unstructured and non-catalytic N-terminal domain helps to search this target substrate by promoting 1D diffusion along the DNA as well as by minimizing unnecessary LIG1 binding to undamaged regions.

## Discussion

Visualizing DNA-binding proteins while interacting with their substrates at the single-molecule level elucidates dynamics of the DNA-binding proteins which are averaged out in bulk assays (60). Single-molecule studies have been extensively applied to examine in real-time how DNA repair proteins detect specific lesions and bind to their targets at extraordinary detail using purified proteins with a fluorescence tag and living cells or cell extracts (61–69). LIG1 is the fundamental enzyme to maintain the structural integrity of the genome as it functions in DNA replication, repair and recombination that all generate strand breaks in the phosphate backbone of DNA (6). As such DNA intermediates can threaten the loss of genetic information and could cause the introduction of deleterious chromosomal mutations if left unrepaired, it's important to understand the details of diverse interactions that LIG1 employs to detect and bind to nick sites on genomic DNA.

The nick sealing activity of LIG1 is attributed to the catalytic region consisting of the AdD and OBD domains that harbor the critical active site residues for catalysis (20). LIG1 protein contains an extended protease sensitive and unstructured N-terminal region that directs the ligase to be recruited to DNA replication foci via a NLS (30). Also, the PIP box motif mediates its interaction with PCNA and is also important for Okazaki fragment synthesis (48–50). LIG1 interacts with many replication and repair proteins through this N-terminal domain (52–54). Furthermore, the serine/threonine residues residing within this region undergo posttranslational modifications and are phosphorylated during cell cycle progression by cyclin-dependent kinase and casein kinase II. It has been shown in mammalian cell models *in vivo* that the N-terminal deletion mutant of LIG1 cannot rescue lethal LIG1-null phenotype and is essential for cellular function and viability to DNA damage inducing agents (32). Although this N-terminal region is dispensable for catalytic activity of LIG1 *in vitro*, its role for nick sensing and binding is largely unknown.

In the present study, we aimed to determine what is central to scanning target nick sites by LIG1 at the single-molecule level. Using combined approaches of C-Trap and TIRF microscope, we visualized the dynamics of LIG1 binding to dsDNA containing ten-, one- or no- nick site in real time. Our C-Trap results demonstrated that after initially binding to DNA, the LIG1 full-length either dissociates fast or exhibits 1D diffusion to search a nick site. Whereas the LIG1 C-terminal mutant predominantly binds non-specifically throughout the DNA and exhibits a diminished ability to diffuse along the DNA to find a nick site. These findings suggest the role of LIG1 N-terminal region in promoting specific nick binding and 1D diffusion along the DNA. Furthermore, a significant percentage of the DNA-bound LIG1 full-length exhibits long-lived residence time on the DNA and LIG1 binding is enriched at nick sites, suggesting the formation of stable DNA-LIG1 complex at the nick sites. For the LIG1 C-terminal protein, the binding lifetimes are significantly shorter without any enrichment at the nick sites, suggesting non-specific and comparatively less stable LIG1 C-terminal/DNA interaction. The C-Trap data were further supported by single-molecule TIRF results that monitor directly LIG1 binding to a DNA with a single nick site at a defined position. Similar long-lived binding events were observed for LIG1 binding to the nick DNA, confirming stable nick binding. Furthermore, the binding lifetimes for LIG1 full-length to DNA without a nick site were significantly short. TIRF results are also consistent with the C-Trap data for C-terminal mutant binding to nick DNA. This also suggests that the short binding events observed during TIRF experiments were due to protein non-specific binding to non-nick regions on the DNA. A plausible explanation for the long-lived LIG1 full-length binding at the nick could be due to the protein spending longer time to undergo necessary conformational changes to encircle the DNA for nick sealing, whereas the shorter binding by C-terminal mutant could be due to the inability of the LIG1 C-terminal protein to efficiently undergo these conformational changes. Together, our findings suggest that the N-terminal domain of LIG1 contributes to an efficient LIG1 binding to nick DNA by first, minimizing non-specific protein binding to non-nick regions and second, by facilitating diffusive behavior of the protein on the DNA to find the nick target and form a stable nick-bound complex.

X-ray and cryo-EM structures of LIG1/PCNA complex reported how PCNA and LIG1 coordinate for efficient nick binding through the N-terminal region (70–72). X-ray structures of ATP-dependent DNA ligase and heterotrimeric PCNA from *Sulfolobus solfataricus* reported that the ligase adopts an open/extended conformation in the absence of nick, and a closed/ring-shaped conformation of the ligase that catalyzes ligation reaction through active site residues residing in the catalytic core is formed when complexed with PCNA, suggesting that this open-to-closed switch in this conformational flexibility of modular three-domain structure of the ligase and PCNA–ligase functional interactions drives and stimulates efficient end joining during multi-step ligation reaction (70). Furthermore, the cryo-EM structure of archaeal DNA ligase and heterotrimeric PCNA in complex with ssDNA break demonstrated a DNA-binding surface formed between the PIP box of DNA ligase and PCNA that supports the distorted conformation of the DNA break undergoing repair and contributes to PCNA stimulation of ligation reaction through contacts at the major (the DBD domain) of the ligase (71). The most recent cryo-EM structure of human LIG1/PCNA complex uncovered that the PIP box serves as an initial tether to PCNA when the ligase is detached from nick and may facilitate the efficient scan of nick sites due to an extremely fast 1D diffusion of PCNA along duplex DNA, which becomes dispensable once the ternary complex of LIG1/PCNA/DNA has formed (72). The study also demonstrated the importance of the DBD domain of LIG1 for efficient ligation and enabling a nick DNA hand off to FEN1 during replication. Our findings of LIG1-nick binding consistent with the studies showing that only the flexible N-terminal domain of LIG1 directs the protein toward PCNA-nick DNA complex, and upon finding a nick site, the catalytic domain of the LIG1 interacts with the DNA and forms a conformationally stable complex at the nick site. Further single-molecule studies will be required to better understand how LIG1 and PCNA coordinate to hand off nick DNA during replication and repair.

Previous single-molecule studies also reported different modes of DNA interactions and collaborative dynamic process during the initial DNA damage search by repair proteins. For example, nucleotide excision repair protein UvrA exhibits 3D diffusion in solution and remains bound on DNA, upon association with UvrB, a significant fraction of UvrA becomes motile and exhibits 1D dissociation (66–69). Similarly, the binding time and diffusion mechanism of DNA glycosylases vary significantly during the damage search process and that an increased binding lifetime suggests that the protein is bound to a damage site (61,62). Poly [ADP-ribose] polymerase 1 (PARP1) has been also reported to exhibit mostly 3D diffusion to identify its substrate and the dissociation of DNA-bound-PARP1 is facilitated in the presence of AP-Endonuclease 1 (APE1) that induces 1D diffusion to enable downstream repair processes (65).

Our study represents a novel insight into the mechanism of nick recognition and binding by LIG1 at the single-molecule level, which is critical to better understand how strand-breaks are repaired at the final ligation step of almost all DNA repair pathways and during the maturation of Okazaki fragments in DNA replication. These insights into nick DNA binding mechanism may potentially lead to the development of effective and specific DNA ligase inhibitors for therapeutic applications (73).

## Supplementary Material

gkae865_Supplemental_File

## Data Availability

The data underlying this article and the MATLAB scripts will be shared on reasonable request to the corresponding author. The MATLAB scripts are available at https://doi.org/10.5281/zenodo.13773762.
