# Supplementary material for: Probing the mechanism of nick searching by LIG1 at the single-molecule level
Source: Nucleic Acids Res. 2024 Oct 15;52(20):12604–15. doi: 10.1093/nar/gkae865 (PMC11551761; doi:10.1093/nar/gkae865)
Supplement: gkae865_Supplemental_File [file gkae865_supplemental_file.pdf]

Supplementary Data

Probing the mechanism of nick searching by LIG1 at the single-molecule level

Surajit Chatterjee[1], Loïc Chaubet[2], Aafke van den Berg[2], Ann Mukhortava[2], Danah Almohdar[1], Jacob Ratcliffe[1], Mitchell Gulkis[1], Melike Çağlayan[1,*]

[1]Department of Biochemistry and Molecular Biology, University of Florida, Gainesville, FL 32610, USA

[2]LUMICKS B.V., 1059 CH, Amsterdam, The Netherlands

*To whom correspondence should be addressed. Tel.: +1 352-294-8383; Email: caglayanm@ufl.edu

Supplementary Figures 1-6

Supplementary Tables 1-5

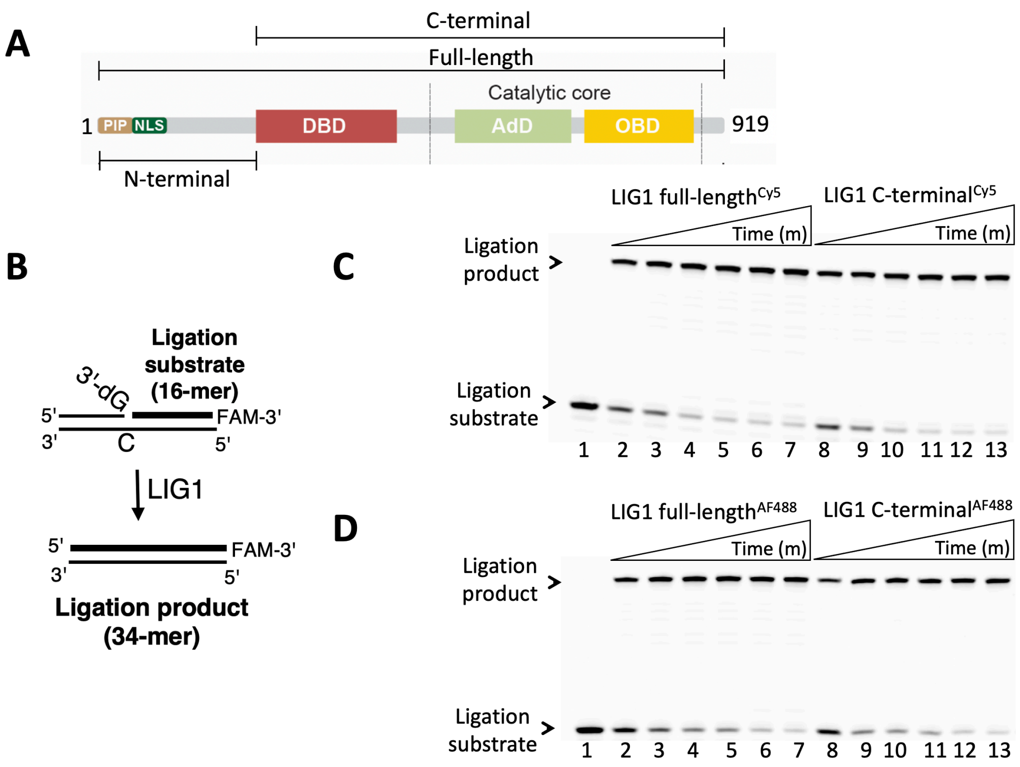

Supplementary Figure 1. (A) The protein domain organization of LIG1. The DNA-binding domain (DBD, red), the catalytic core consisting of the adenylation (AdD, green) and Oligonucleotide-binding (OBD, yellow) domains and the N-terminal domain including the nuclear localization signal (NLS) and proliferating cell nuclear antigen interacting peptide (PIP) box are indicated. **(B)** Scheme shows the nick DNA substrate with 3'-FAM label and ligation product observed in the ligation assays including LIG1. **(C)** Line 1 is the negative enzyme control of nick DNA substrate with 3'-dG:C. Lanes 2-7 and 8-13 are the nick sealing products by LIG1 full-length and C-terminal proteins, respectively, and correspond to time points of 0.5, 1, 3, 5, 8, and 10 min, after Cy5 labeling to use in the TIRF single-molecule experiments. **(D)** Line 1 is the negative enzyme control of nick DNA substrate with 3'-dG:C. Lanes 2-7 and 8-13 are the nick sealing products by LIG1 full-length and C-terminal proteins, respectively, and correspond to time points of 0.5, 1, 3, 5, 8, and 10 min, after AF488 labeling to use in the C-Trap single-molecule experiments.

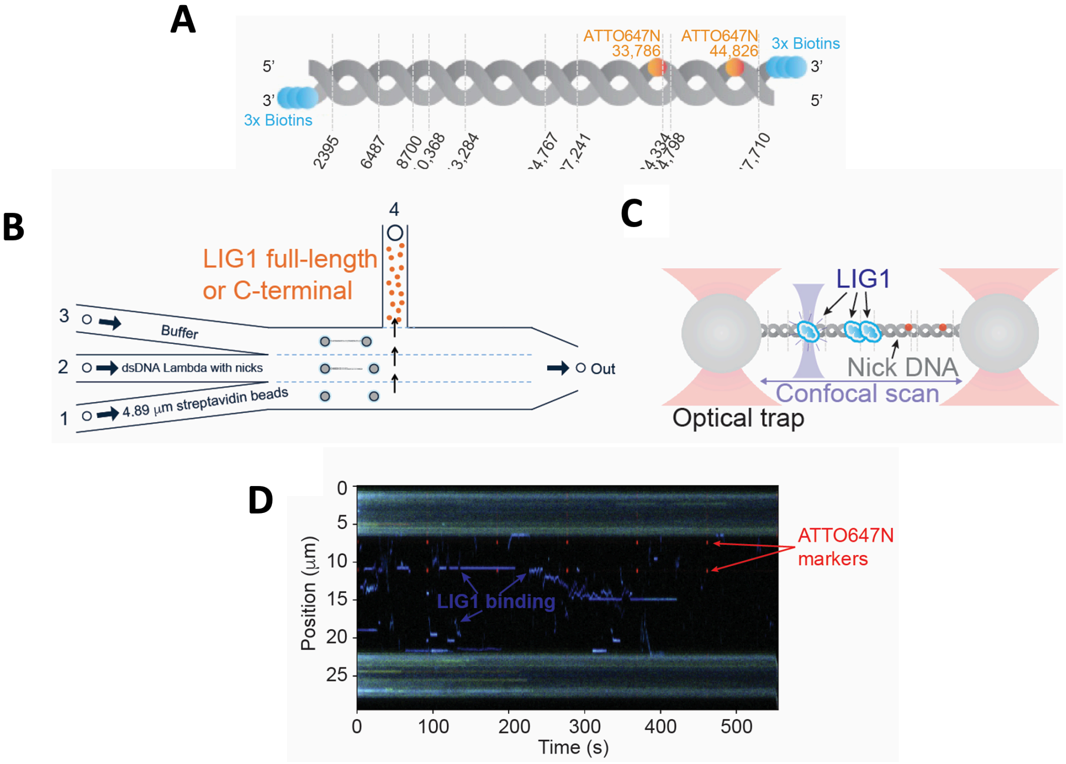

Supplementary Figure 2. (A) Schematic representation of the 48,502 bp biotinylated dsDNA with ten nicks used in the C-Trap single-molecule experiments. The two fluorophores (ATTO647N) at position 33,786 bp and 44,826 bp were used to identify the locations of the nicks. **(B)** Scheme describes the workflow of the C-Trap instrument combining three-color confocal fluorescence microscopy with dual-trap optical tweezers (LUMICKS). A microfluidic flow-cell containing four distinct flow channels separated by laminar flow was moved by a computer-controlled stage to allow two optical traps to traverse the different laminar layers. Channels 1 and 2 are filled with 4.89 μm streptavidin-coated polystyrene beads and biotinylated lambda dsDNA, respectively. Channel 3 is used for trap calibration in the reaction buffer and channel 4 contains AF^{488}-labeled LIG1 (full-length or C-terminal). When a single DNA tether was confirmed, the traps were moved into the protein channel 4 where confocal scanning of LIG1 on DNA was measured. **(C)** Scheme shows the DNA captured between two optically trapped polystyrene beads for confocal scanning

of AF^{488-}labeled LIG1 binding on nick DNA. **(D)** Example of kymograph displays the ATTO647N markers and AF^{488}-labeled LIG1 full-length protein (blue) binding events on the DNA as a function of time. The markers (ATTO647N) are first located using a peak detection algorithm on the red channel and then the known coordinates of the markers were used to identify the locations of the nicks.

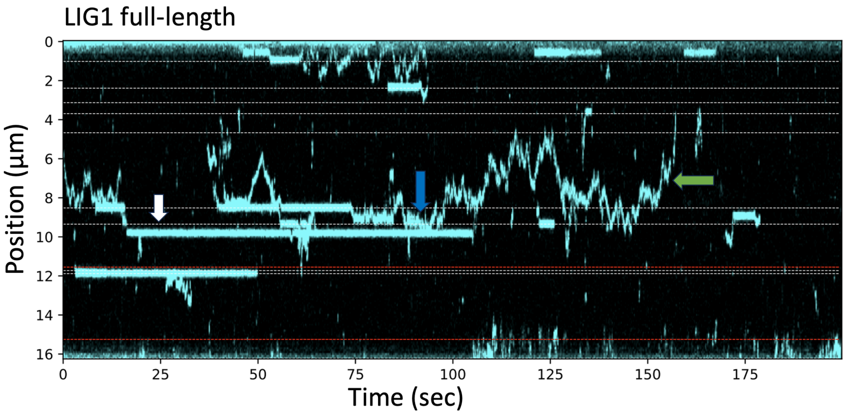

Supplementary Figure 3. Example of kymograph shows the single molecule dynamics of AF^{488}-labeled LIG1 full-length protein binding on nick DNA as a function of time. LIG1 occasionally shows stable binding on a location that is not one of the predicted nicks (white arrow). The diffusive ligase cannot pass static ligase (blue arrow) and is capable of 1D diffusion past several predicted nick sites (green arrow).

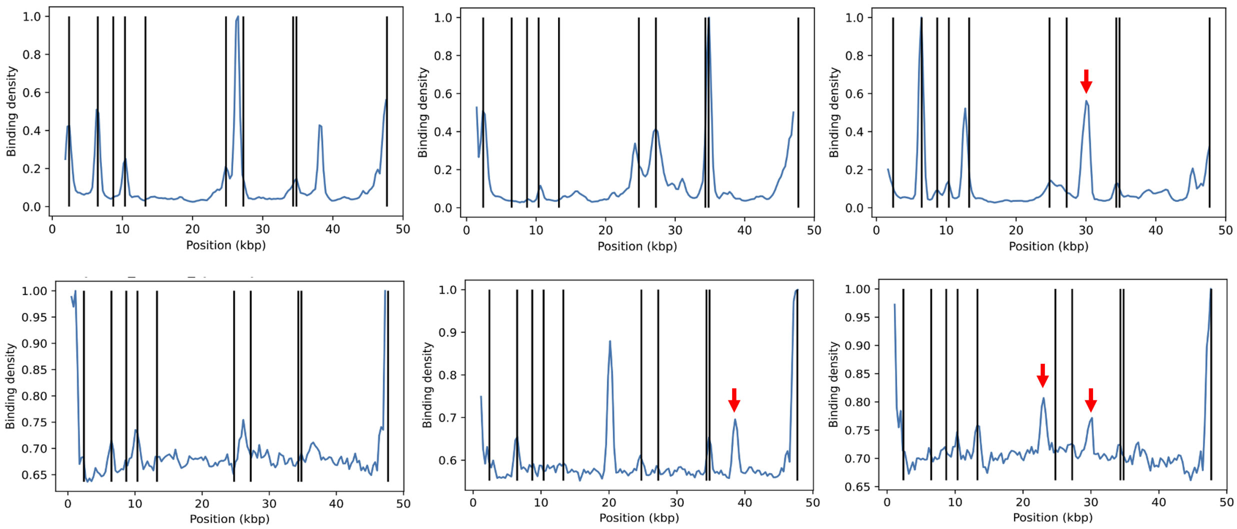

Supplementary Figure 4. Nick DNA binding kinetics of LIG1 full-length. DNA binding profiles for individual kymographs for 50 sec duration/each of LIG1 full-length. Peaks (blue) correspond to the ligase bound to DNA and the vertical lines (black) represent nick coordinates and their positions on dsDNA substrate (kbp). Red arrows indicate non-specific and off-target DNA bindings by LIG1. These off-target binding sites could correspond to binding to unpredicted nick sites. Overlaps of peaks with vertical lines correspond to LIG1 bound to one of the ten predicted nick sites on dsDNA.

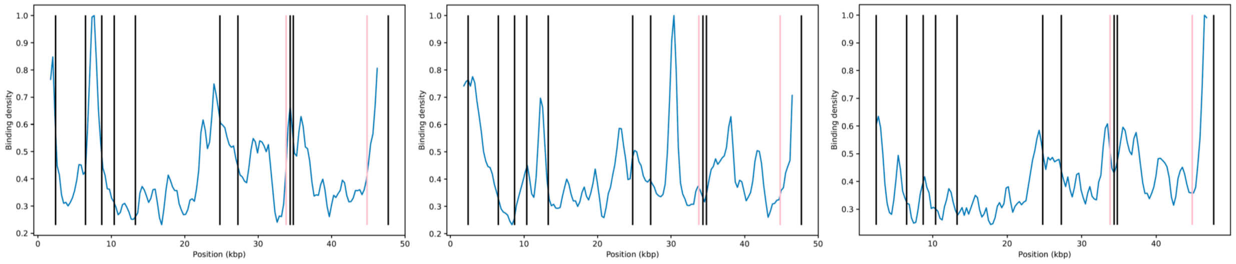

Supplementary Figure 5. Nick DNA binding kinetics of LIG1 C-terminal protein. DNA binding profiles for individual kymographs for 50 sec duration/each of LIG1 C-terminal protein. Peaks (blue) correspond to the ligase bound to DNA and the vertical lines (black) represent nick coordinates and their positions on dsDNA substrate (kbp).

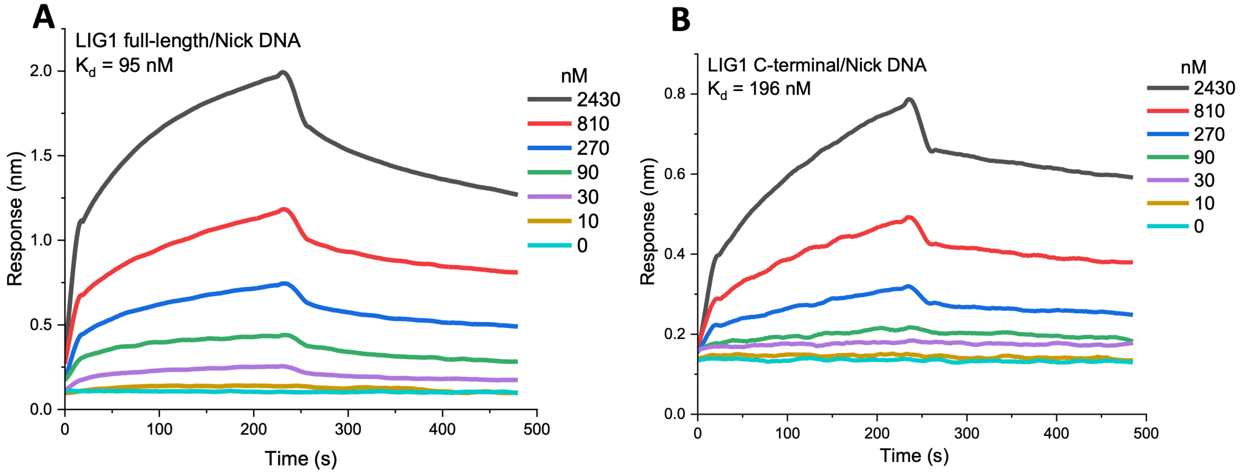

Supplementary Figure 6. Nick DNA binding affinities of LIG1 proteins by BLI assay. Nick DNA binding kinetics was measured by BLI assay, and the equilibrium binding constants (K_D) are shown for LIG1 full-length (A) and C-terminal (B) proteins. Sensorgrams are shown for the concentrations range of the proteins where the nick DNA with a biotin label is immobilized on the streptavidin biosensor.

Protein	Labeling Efficiency
LIG1 full-length^{AF488}	1.7
LIG1 C-terminal^{AF488}	1.3
LIG1 full-length^{Cy5}	0.9
LIG1 C-terminal ^{Cy5}	1.4

Supplementary Table 1. Labelling efficiencies of LIG1 proteins. LIG1 proteins were labeled with amine reactive NHS ester-conjugated AF-488 and Amersham Cy5 monoreactive NHS ester dye to use in C-Trap and TIRF single-molecule experiments, respectively.

Nick DNA Substrate	Sequence
3'-dG:C	5'-CATGGGCGGCATGAACC**G**GAGGCCCATCCTCACC-3'-FAM<br>3'-GTACCCGCCGTACTTGGCCTCCGGGTAGGAGTGG-5'
3'-dG:C/5'-Bio	Bio-5'-CATGGGCGGCATGAACC**G**GAGGCCCATCCTCACC-3'<br>3'-GTACCCGCCGTACTTGGCCTCCGGGTAGGAGTGG-5'

Supplementary Table 2. Nick DNA substrate used in ligation and BLI assays. FAM denotes a fluorescence tag and is located at the 3'-end. Bio denotes a Biotin label located at 5'-end. The base at 3'-end is shown as bold and the template base is underlined. Nick DNA substrate with 3'-dG:C was used for ligation assays to test the nick sealing efficiency of LIG1 full-length and C-terminal proteins after labeling. Nick DNA substrate 3'-dG:C/5'-Bio was used for BLI assays to test the nick DNA binding affinity of LIG1 full-length and C-terminal proteins.

Oligonucleotide	Sequence
Up-OH	5'-Bio-CATGGGCGGCATGAACC**A**-3'
Up-ddC	5'-Bio-CATGGGCGGCATGAACC**X**-3'
Template T	5'-GGTGAGGATGGGCCTCTGGTTCATGCCGCCCATG-3'
Template G	5'-GGTGAGGATGGGCCTCGGGTTCATGCCGCCCATG-3'
Down	5'-(P)GAGGCCCATCCTCACC-AF488-3'
Top	5'-Bio-CATGGGCGGCATGAACC**A**GAGGCCCATCCTCACC-AF488-3'

Supplementary Table 3. Nick DNA substrates used in the single molecule characterization of LIG1/nick DNA binding in the TIRF. Bio denotes a Biotin label located at 5'-end, AF488 is a green-fluorescent dye located at 3'-end, and P stands for a Phosphate at 5'-end. The base at 3'-end is shown as bold and the template base is underlined. Up-OH, Template T, and down oligonucleotides were used to prepare the DNA substrate with a single nick site. Top and Template T oligonucleotides were used to prepare double-strand DNA without nick site. Up-ddC, Template G, and down oligonucleotides were used to prepare the DNA substrate with a single nick site and non-ligatable ends. X denotes 3'-ddC modification.

LIG1	DNA	t_{bound}, s (amplitude)	$t_{unbound}$, s (amplitude = 1)	*N*
Full-length	Nick	0.9 ± 0.17 (0.7 ± 0.07)<br>8 ± 0.6 (0.3 ± 0.07)	42 ± 4	335
C-terminal	Nick	0.8 ± 0.2 (0.8 ± 0.1)<br>3.5 ± 0.4 (0.2 ± 0.1)	86 ± 10	313

Supplementary Table 4. Single-molecule analyses of LIG1/DNA binding in the TIRF. Protein-bound and unbound lifetimes extracted from the single-molecule fluorescence colocalization experiments are presented for LIG1 full-length and C-terminal proteins for DNA substrate containing a single nick site. Values represent the average ± standard deviation of the mean from three independent experiments. N is the total number of analyzed molecules.

DNA	t_{bound}, s (amplitude)	$t_{unbound}$, s (amplitude = 1)	*N*
Nick<br>with ligatable ends<br>(No Mg^{2+})	0.9 ± 0.17 (0.7 ± 0.07)<br>8 ± 0.6 (0.3 ± 0.07)	42 ± 4	335
Nick<br>with non-ligatable ends<br>(3'-ddC modification)	1±0.1 (0.6±0.1)<br>7±1.5 (0.4±0.1)	52±1	252
Nick<br>with ligatable ends<br>(+ Mg^{2+})	0.3±0.01 (0.8±0.03)<br>2.9±0.14 (0.2±0.03)	136±19	175
No nick	0.26 ± 0.04 (0.8 ± 0.02)<br>2.8 ± 0.5 (0.2 ± 0.02)	98 ± 6	286

Supplementary Table 5. Single-molecule analyses of LIG1/DNA binding in different conditions. Protein-bound and unbound lifetimes extracted from the single-molecule fluorescence colocalization experiments are presented for LIG1 full-length protein. Single-molecule experiments were performed using nick DNA substrates with a single nick site in the absence and presence of Mg^{2+} or using the DNA substrate containing no nick site as well as the nick DNA substrate with non-ligatable ends due to the 3'-ddC modification. Values represent the average ± standard deviation of the mean from three independent experiments. *N* is the total number of analyzed molecules.
